# Arsenical keratosis in China: A case report and review of the literature

**DOI:** 10.1111/srt.13903

**Published:** 2024-08-27

**Authors:** Rong Tao, Ruojun Wang

**Affiliations:** ^1^ Department of Dermatology Beijing Tongren Hospital Capital Medical University Beijing China; ^2^ Department of Dermatology Peking University First Hospital National Clinical Research Center for Skin and Immune Diseases, Beijing Key Laboratory of Molecular Diagnosis on Dermatoses, NMPA Key Laboratory for Quality Control and Evaluation of Cosmetics Beijing China

**Keywords:** arsenic poisoning, arsenical keratosis, traditional Chinese medicine

## Abstract

**Background:**

Arsenical keratosis is a precancerous dermatosis which could be induced by long‐term exposure to arsenic poisoning. Arsenic is often added to traditional Chinese medicine in a non‐compliant manner to increase the effectiveness of psoriasis treatment, which is often the main cause of arsenic poisoning in Chinese patients with psoriasis.

**Objectives:**

We performed a systemic review of arsenic keratosis during the past 32 years to better understand the sources, treatment, and prognosis of arsenic keratosis in China.

**Methods:**

We searched Medline/PubMed, Embase, CNKI, and Wanfang databases for research studies published between 1992 and 2024. A total of 64 papers with 78 individual Chinese of arsenical keratosis were included in this analysis.

**Results:**

Of the patients included in the analysis, 92.21% of arsenic poisoning was due to iatrogenic factors: Chinese traditional medicine. Seventy‐six patients (98.70%) had skin manifestation of hyperkeratotic papules and plaques, 68 patients (88.31%) had hyperpigmentation, 43 cases (55.84%) had hypopigmentation, and only 4 had a clear indication of Mees' lines in nails. A total of 52.63% of patients presented with tumors, including squamous cell carcinoma, Bowen's disease, and basal cell carcinoma. For patients with tumors, 20 opted for surgery, 6 for radiotherapy, and 3 for PDT. All patients with only cutaneous tumors are currently well‐controlled. Death occurred in one patient with metastatic squamous cell carcinoma. Keratinizing papules improved significantly in 70.59% of patients treated with Acitretin Capsules.

**Conclusions:**

In this study, arsenic sources in Chinese patients were mainly from traditional Chinese medicine, and there were no reports of exposure to water sources or occupational sources in the past 32 years. Most of the patients showed keratinizing papules and pigmentation, and more than 1/2 of the patients showed skin tumors, mainly squamous cell carcinoma. The treatments of tumors are mainly surgical treatment, PDT and radiotherapy can also be selected. The improvement in keratinizing rash was greater than 70% with acitretin capsules. Patients with this disease should be regularly followed up for early detection and timely treatment of potential malignant tumors.

## 1 INTRODUCTION

Arsenical keratosis is a precancerous dermatosis which could be induced by long‐term exposure to arsenic poisoning. The common sources of arsenic toxicity include contaminated groundwater, medications, occupational exposure, and tobacco.^1^ Although iatrogenic chronic arsenic poisoning is seldom reported in Western countries, it can be occasionally encountered in clinics in China. Realgar, also called arsenic sulfide, has been widely used in combination with other herbs for the treatment of many diseases, including psoriasis, in traditional Chinese medicine. Other causes of arsenical keratosis are uncommonly reported in China, and water‐borne arsenic poisoning can be traced back to the 1980s in Xinjiang, Qinghai, Ningxia, and Inner Mongolia regions.^2^

The main clinical manifestations of arsenical keratosis are corn‐like yellowish hyperkeratotic papules and plaques, primarily affecting the palms and soles. It can also cause skin hyperpigmentation and Mees' lines in nails. In patients with long‐term diseases, the lesions can spread to other parts of the body and even lead to malignancies of the skin, lungs, liver, urinary tract, and other organs.^3^ As the earliest and most common presenting complaint, arsenical keratosis can help guide clinicians, especially dermatologists, toward the early diagnosis and management of chronic arsenic toxicity.^1^

Here, we reported a patient who developed arsenic keratosis after long‐term use of traditional Chinese medicine for psoriasis. Considering the high utilization rate of traditional Chinese medicine in Chinese population, we performed a systemic review of arsenic keratosis to better understand the sources, clinical features, treatment, and prognosis of arsenic keratosis during the past 32 years in China.

## 2 CASE REPORT

**FIGURE 1:**
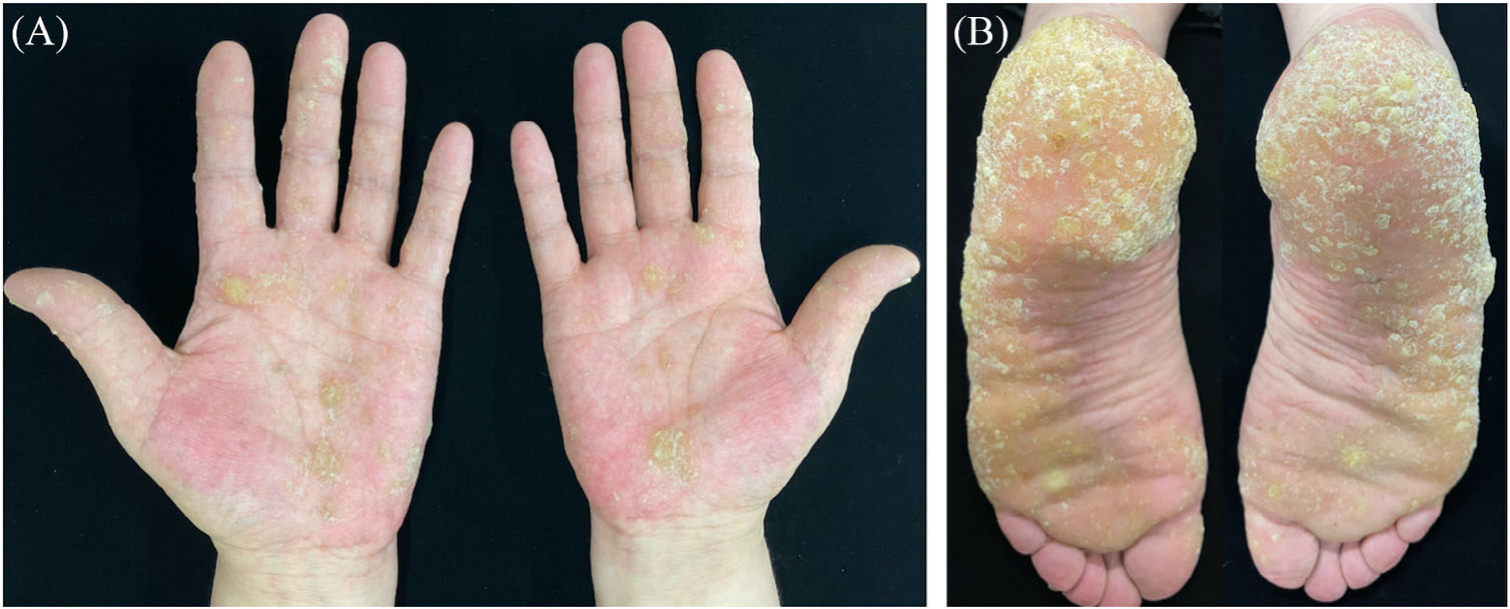
(A and B) Clinical image showed diffuse verrucous, yellowish, hyperkeratotic papules, and plaques affecting bilateral palms, soles, and the fingers.

**FIGURE 2:**
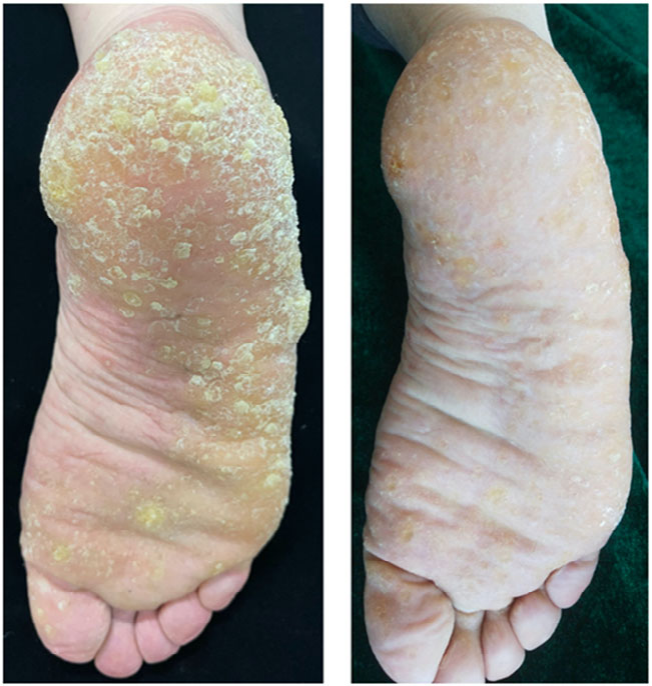
The hyperkeratotic papules significantly improved 3 months later.

A 51‐year‐old man presented with a 7‐year history of asymptomatic keratinizing skin lesions (Figure 1A, B). The patient had a 20‐year history of psoriasis, for which he began to take realgar and other herbs 9 years ago. He stopped the herbs 1 year later due to hyperkeratosis of the palms. On physical examination, diffuse verrucous, yellowish, hyperkeratotic papules, and plaques were observed on bilateral palms, soles, and fingers. The patient was otherwise healthy and denied a family history of similar symptoms. Laboratory tests for the liver and renal functions were within normal range. Screening tests for tumor markers showed no abnormalities. A diagnosis of arsenical keratosis was made based on his medical history and typical clinical manifestations. The patient was administered oral acitretin 20 mg per day. The skin lesions significantly improved 3 months later, and he is currently on a close follow‐up visit (Figure 2).

## 3 MATERIALS AND METHODS

### 3.1 Literature search

We conducted a systematic search of PubMed, Embase, and two Chinese databases, including the China Knowledge Resource Integrated (CNKI) database (http://www.cnki.net) and the Wanfang database (http://www.wanfangdata. com.cn/index.html) using the term “arsenic keratosis.” Papers published between 1992 and 2024 were included. References of included papers were checked and screened. Two authors (TR and WRJ) searched and screened the eligible papers independently. Review papers, conference abstracts, non‐Chinese patients, and unrelated articles were excluded. The screening flow chart is shown in Figure S1.

### 3.2 Data extraction

The following data were extracted: first author, publication year, living areas, sex, age, arsenic sources, length of using arsenic agent, accompanied diseases, skin manifestations, secondary tumors, treatment, and outcome.

## 4 RESULTS

A total of 64 papers with 78 cases of arsenical keratosis published between 1992 and 2024 in China were identified.^4^, ^5^ Coupled with the case we report here, a total of 76 patients were included in the study. The average age of the patients was 44.01 ± 16.82 years (range: 10–90 years), and the male to female ratio was 57:21. Patients’ demographics, sources of arsenic, clinical manifestations, treatment, and outcomes are presented in Tables 1 and 2. More detailed information can be found in Table S1.

**TABLE 1:** Patients’ demographics and clinical manifestations.

	Patients with tumors (*n* = 41)	Patients without tumors (*n* = 37)
Mean age (years, mean ± SD)	54.24 ± 12.44	32.36 ± 13.24
Sex (M:F ratio)	32:9	25:12
Accompany diseases		
Psoriasis vulgaris	24	23
Asthma	7	0
Epilepsy	2	4
Others	2	9
None	6	1
Time exposure to arsenic (years, mean ± SD)	7.88 ± 9.90	3.88 ± 6.05
Skin manifestations		
Hyperkeratotic papules	40	36
Hyperpigmentation	33	35
Hypopigmentation	19	24
Mees' lines in nails	2	2

**TABLE 2:** The treatment and outcomes of patients.

Treatment		Cured	Improved	Loss to follow‐up	Total
Arsenic removal agents	Dimercaptopropyl sulfonate	4	6	4	14
	Sodium thiosulfate	1	2	5	8
	Others			2	2
Retinoic acid	Without tumors		4	1	5
	With tumors		9	6	15
Treatment of tumors	Operation	5		10 (one death)	15
	Operation + PDT	2			2
	Operation + radiotherapy		1	1	2
	Operation + Imiquimod cream	1	1		2
	Radiotherapy	3		1	4
	PDT	1			1
	Imiquimod cream		1		1
	5‐Fluorouracil	1	1	1	3

Abbreviation: PDT, photodynamic therapy.

### 4.1 Sources of arsenic exposure

Seventy‐one (92.21%) of the cases were caused by iatrogenic arsenic poisoning due to traditional Chinese medicine. Realgar was the most common causal factor (30 cases), followed by arsenic (8 cases) and arsenic‐containing compounds (4 cases). Psoriasis vulgaris was the most commonly reported disease that needed arsenic as a therapy in traditional Chinese medicine (47cases, 66.20%), followed by epilepsy (6 cases, 8.45%), asthma (7 cases, 9.86%), constipation (2 cases, 2.82 %), and dermatitis (2 cases, 2.82%). Other unusual medical histories included glomerulonephritis, superficial fungal infections, depression, ulcerative colitis, rheumatoid arthritis, vitiligo, and myeloid leukemia. One case reported using combined realgar and arsenic for the treatment of constipation before the occurrence of arsenic keratosis. In addition to iatrogenic reasons, arsenic poisoning was caused by drinking groundwater in 3 cases, occupational exposure in 2 cases of mine workers, and accidental ingestion of arsenic‐contaminated food in 2 cases. Some areas in Bayannur district of Inner Mongolia and Shandong Province were reported to have high level of arsenic concentration in the groundwater. More detailed information can be found in Table S1.

### 4.2 Clinical manifestations

The skin manifestations of arsenical keratosis included hyperkeratotic papules and plaques (76 cases, 98.70%), hyperpigmentation (68 cases, 88.31%), hypopigmentation (43 cases, 55.84%), and Mees' lines in nails (4 cases, 5.26%). The average time between exposure to arsenic agent and occurrence of skin symptoms was 3.64 ± 5.73 years (Table 1). A total of 41 (52.63%) patients presented with malignancies, and the most common malignancy was squamous cell carcinoma (31 cases), followed by Bowen's disease (19 cases), and basal cell carcinoma (2 cases). The average time between arsenic exposure to the occurrence of tumor was 19.10 ± 12.85 years. Three patients had metastatic squamous cell carcinoma: one case presented with bone metastasis that developed 24 years after arsenic exposure, and two cases had lymph node and oral metastasis. Since the arsenic content was not provided in most medications, we were not able to confirm the existence of dose‐dependent side effects.

Other arsenical‐associated symptoms included hematologic changes in five patients (three had anemia, one had thrombocytopenia, and one had myelodysplastic syndrome), abnormal liver function in four patients (including one developed cirrhosis), numb or insensitive in two patients, and fatigue and anorexia in three patients. A single patient presented with self‐reported symptoms of blurred vision, lacrimation, and photophobia. More detailed information can be found in Table S2.

### 4.3 Treatment and outcomes

Arsenic removal agents, including dimercaptopropyl sulfonate and sodium thiosulfate, were widely used for the treatment of arsenic poisoning in China. These medications were administered in 18 (43.90%) patients without malignancies and 6 (14.63%) patients with malignancies, resulting in significantly decreased blood and urine arsenic concentrations. The blood arsenic levels significantly decreased in 2 patients (25.00%) receiving sodium thiosulfate and returned to normal levels in 4 patients (44.44%) treated with dimercaptopropyl sulfonate. However, two patients with malignancies showed elevated urinary arsenic levels after treatment with dimercaptopropyl sulfonate. Retinoic acid was administered in 20 patients (50.0%), including 15 patients with tumors. This medication significantly improved keratinizing papules in 13 of the patients (65.00%).

For those with secondary tumors, 20 patients opted for removal surgery, 6 for radiotherapy, and 3 for photodynamic therapy (PDT). One patient underwent surgery after failing PDT, and another 2 patients were treated with a combined surgery with PDT or radiotherapy for multiple tumors. Four patients with Bowen's disease showed significant improvement with topical 5‐fluorouracil or imiquimod cream alone. All patients with solely cutaneous tumors responded well; however, death occurred in one patient with metastatic squamous cell carcinoma (Table 2). More detailed information can be found in Table S2.

## 5 DISCUSSION

Arsenical keratosis is a precancerous dermatosis induced by long‐term exposure to arsenic poisoning. According to the World Health Organization, arsenic poisoning is defined as the ingestion of arsenic agents, a class I human carcinogen, exceeding safe limits for at least 6 months.^6^ In China, arsenical keratosis is rarely encountered in clinical practice due to the wide use of realgar (As2S2) and arsenic (AS2O3) for the treatment of many diseases in traditional Chinese medicine. In this study, we performed a systemic review of arsenic keratosis to better understand the arsenic exposure sources, clinical manifestations, treatment, and prognosis of arsenic keratosis in China.

Contaminated groundwater, food crops, medications, occupational exposure, and tobacco have been considered the major sources of arsenic toxicity. China was once thought to have a high incidence of arsenic poisoning due to the presence of groundwater with excessive levels of arsenic, but the situation has changed in the past 30 years. Before the 1980s, a small proportion of arsenic poisoning cases were caused by occupational exposure and exposure to water sources. After the 1990s, the Chinese government strengthened investment in occupational protection and drinking water projects, and no related arsenic exposure has been reported.

Different from other countries, iatrogenic arsenic poisoning has been occasionally reported in the last decades due to the wide use of arsenic in China. Realgar is effective for the treatment of psoriasis through inhibiting human keratinocyte proliferation by the induction of apoptosis.^7^, ^8^ This medication was also reported to have therapeutic effects for acute promyelocytic leukemia and MDS by reducing DNA hypermethylation.^9^ Despite its therapeutic effects, the long‐term toxic effects of arsenic have raised concern in Chinese medicine specialists, and corresponding adjustments have been made to the clinical usage of realgar. However, our review showed that all the iatrogenic cases had a long history of using drugs without regular follow‐up visits, and 70% of the patients were treated by informal Chinese medicine doctors. Since the contents of medications are not standardized in prescriptions, it is difficult to quantify the arsenic content and assess drug toxicity dose.

Chronic arsenic poisoning increases the incidence of cancers, but its exact pathogenesis in inducing premalignant and malignant is not known. Current studies suggest that arsenite methyltransferase (AS3MT), the main factor mediating the methylation process, may lead to depletion of S‐adenosine and hypomethylation of the entire DNA, causing abnormal gene expression in cells and predisposes to cancer.^10^, ^11^ The reactive oxygen species produced in the process of arsenic metabolism can also lead to DNA oxidative damage, chromosomal abnormalities and increase the risk of cancer.^10^, ^11^ Based on previous studies, only 15%–20% of skin lesions occurred under arsenic exposure, which is further confirmed by an individual's genetic susceptibility.^11^ In this analysis, more than half of the patients developed skin tumors, usually cutaneous squamous cell carcinoma. The high rate of malignant transformation might be due to publication bias with severe patients more likely to be reported.

The diagnosis of arsenic keratosis is not difficult, mainly based on the typical clinical manifestations and if necessary, histological examination to exclude malignant transformation. Hyperkeratosis, parakeratosis, and acanthosis are the normal features of arsenical keratosis in the examination of histopathology.^12^ When a diagnosis is confirmed, active search for the source of arsenic and stopping further exposure is the first and foremost thing for treatment. Sodium thiosulfate can decrease arsenic levels in the blood, making it a common removal agent in clinical practice.^13^ Supplements of antioxidants such as vitamins A, C, and E are also thought to protect against cancers.^10^ Oral acitretin, combined with topical keratolytic agents, can be used to treat patients with extensive lesions and used for chemoprevention of cutaneous malignancies.^14^ In our analysis, approximately 70% keratinizing skin lesions were improved after using oral acitretin, indicating this medication as an effective choice for eliminating hyperkeratotic lesions in arsenic keratosis. For patients with skin malignancies, Mohs surgery or expanded excision is recommended; and other therapies include photodynamic, cryotherapy, and topical imiquimod.^1^ Due to the potential risk of malignant transformation, patients should be regularly followed up for early detection and timely treatment of malignancies.

During our screening process, we identified three cases of arsenic keratosis due to taking traditional Chinese medicine reported by Korean doctors. All patients had concomitant neoplasms, including Bowen's disease, cutaneous squamous cell carcinoma, with one patient having multiple Bowen's disease and secondary genitourinary tumors.^12^, ^13^, ^14^ This suggest that doctors in countries other than China should be aware of this disease when evaluating cutaneous lesions that present with multiple hyperkeratotic lesions and hyperpigmentation.

## 6 CONCLUSION

In this study, we reviewed cases of arsenic keratosis in China and showed that iatrogenic arsenic poisoning was the main cause of this disorder. Keratinizing papules and pigmentation are the most common manifestations, and approximately one in a third of the patients developed skin tumors. Clinicians should have an acute awareness of this disease and actively search for the source of arsenic poisoning. Patients should be regularly followed up for early detection and timely treatment of potential malignancies.

## CONFLICT OF INTEREST STATEMENT

The authors declare no conflicts of interest.

## CONSENT STATEMENT

The patients in our case study have signed the informed consent forms.

## Supporting information

Supporting Information

Supporting Information

## Data Availability

The data that supports the findings of this study are available in the supplementary material of this article.
