# Correction to “Arsenical Keratosis in China: A Case Report and Review of the Literature”

**DOI:** 10.1111/srt.70275

**Published:** 2025-10-09

R. Tao, and R. Wang, “Arsenical Keratosis in China: A Case Report and Review of the Literature,” *Skin Research and Technology* 30, no. 9 (2024): e13903, https://doi.org/10.1111/srt.13903.

The original version of the Supporting Information file, which included Tables S1 and S2, omitted the complete list of references used in the literature review.

To address this omission, the Supporting Information file has been replaced with an updated version that includes the complete list of references. As many of the cited articles were published in Chinese journals, the list now contains both the original references in Chinese and their English translations.

The authors apologize for these omissions and for any inconvenience they may have caused.

