# Supplementary figures and images for: Arsenical keratosis in China: A case report and review of the literature
Source: Skin Res Technol. 2024 Aug 27;30(9):e13903. doi: 10.1111/srt.13903 (PMC11348506; doi:10.1111/srt.13903)

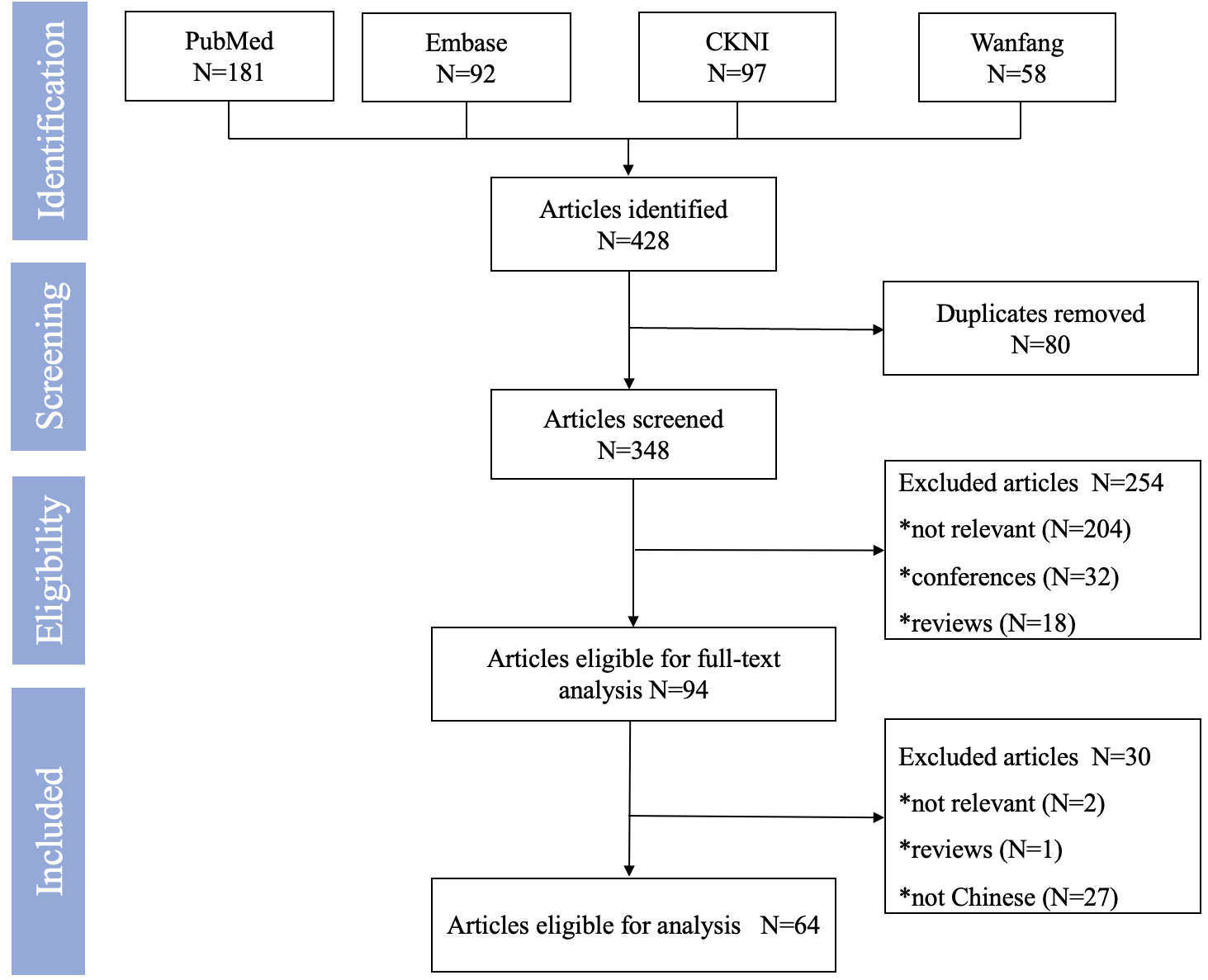

Supplement: Supplementary file 1 — Supporting Information [file SRT-30-e13903-s002.png]
