# Supplementary material for: Arsenical keratosis in China: A case report and review of the literature
Source: Skin Res Technol. 2024 Aug 27;30(9):e13903. doi: 10.1111/srt.13903 (PMC11348506; doi:10.1111/srt.13903)
Supplement: Supplementary file 2 — Supporting Information [file SRT-30-e13903-s001.docx]

STable 1: The patients’ demographics and sources of arsenic

| Author | Year | Areas | Gender | Age (years) | Sources | Use Time（years） | Diseases |
| --- | --- | --- | --- | --- | --- | --- | --- |
| The paper | 2024 | China | M | 51 | Traditional Chinese medicine | 1 | Psoriasis |
| Manli Sun et al | 2024 | China | M | 34 | Traditional Chinese medicine | 0.58 | Psoriasis |
| Weiyi Li et al | 2023 | China | M | 60 | Traditional Chinese medicine | 15 | Psoriasis |
| Juanjuan Wang et al | 2022 | China | M | 19 | Traditional Chinese medicine | 2 | Psoriasis |
| Zhaorui Liu et al | 2021 | China | M | 68 | Traditional Chinese medicine | 3 | Psoriasis |
| Xianxu Yang et al | 2021 | China | M | 54 | Traditional Chinese medicine | 2 | Asthma |
| Cuicui Tian et al | 2021 | China | M | 55 | Traditional Chinese medicine | UK | Asthma |
| Cuicui Tian et al | 2021 | China | F | 60 | Traditional Chinese medicine | UK | Asthma |
| Cuicui Tian et al | 2021 | China | M | 94 | Traditional Chinese medicine | UK | Asthma |
| Ying Li et al | 2019 | China | M | 60 | Traditional Chinese medicine | 2 | Psoriasis |
| Qifeng Wang et al | 2018 | China | M | 70 | Occupational exposure | NK | No |
| Zaiyong Wu et al | 2018 | China | M | 55 | Traditional Chinese medicine | NK | Psoriasis |
| Bingmei Liu et al | 2018 | China | M | 22 | Traditional Chinese medicine | 8 | Psoriasis |
| Bingmei Liu et al | 2018 | China | M | 28 | Traditional Chinese medicine | 5 | Psoriasis |
| Jingqiu Zhang et al | 2017 | China | M | 19 | Traditional Chinese medicine | 4 | Psoriasis |
| Yanping Huang et al | 2017 | China | M | 52 | Groundwater | 29 | No |
| Yang Li et al | 2016 | China | F | 51 | Traditional Chinese medicine | NK | Psoriasis |
| Xiaoqin Yi et al | 2016 | China | F | 43 | Traditional Chinese medicine | 2 | Psoriasis |
| Hongguo Li et al | 2016 | China | F | 52 | Traditional Chinese medicine | 7 | Psoriasis |
| Jianfeng Yang et al | 2015 | China | M | 72 | Traditional Chinese medicine | 3 | Psoriasis |
| Xiuling Liu et al | 2014 | China | M | 69 | Traditional Chinese medicine | 1.5 | Asthma |
| Xiaowen Chen et al | 2014 | China | M | 39 | Traditional Chinese medicine | 1.5 | Psoriasis |
| Xiaowen Chen et al | 2014 | China | M | 36 | Traditional Chinese medicine | 3 | Psoriasis |
| Xiaowen Chen et al | 2014 | China | M | 24 | Traditional Chinese medicine | 8 | Glomerulonephritis |
| Sijing Zhou et al | 2014 | China | M | 70 | Traditional Chinese medicine | 1/6 | Dermatitis |
| Sijing Zhou et al | 2014 | China | F | 42 | Traditional Chinese medicine | 1/4 | Dermatitis |
| Yajuan Ji et al | 2013 | China | M | 23 | Traditional Chinese medicine | 2 | Psoriasis |
| Rina Wu et al | 2013 | China | M | 60 | Groundwater | 18 | No |
| Xiaojuan Nie et al | 2012 | China | F | 45 | Groundwater | 1 | No |
| Cuiping Gao et al | 2012 | China | M | 43 | Traditional Chinese medicine | 6 | Psoriasis |
| Hua Zhang et al | 2012 | China | M | 51 | Traditional Chinese medicine | 1.5 | Psoriasis |
| Jielin Zhang et al | 2012 | China | F | 44 | Traditional Chinese medicine | 0.25 | tinea of feet and hands |
| Wei Zhao et al | 2011 | China | M | UK | Traditional Chinese medicine | 2 | depression |
| Yuping Chen et al | 2011 | China | M | 61 | Traditional Chinese medicine | 1 | Psoriasis |
| Ruili Zhang et al | 2011 | China | M | 57 | Traditional Chinese medicine | 1 | Asthma |
| Jun Wang et al | 2011 | China | M | 46 | Traditional Chinese medicine | UK | Psoriasis |
| Biao Zhang et al | 2011 | China | F | 32 | Traditional Chinese medicine | 1 | Psoriasis |
| Biao Zhang et al | 2011 | China | F | 33 | Traditional Chinese medicine | 1.5 | Ulcerative colitis |
| Xiaoxia Liu et al | 2010 | China | M | 65 | Traditional Chinese medicine | 10 | Asthma |
| Wei Zhu et al | 2010 | China | M | 21 | Traditional Chinese medicine | 1/6 | Psoriasis |
| Guangcheng Gao et al | 2010 | China | M | 62 | Traditional Chinese medicine | UK | Psoriasis |
| Yuan Lu et al | 2010 | China | M | 21 | Traditional Chinese medicine | 1 | Psoriasis |
| Jin Wei et al | 2010 | China | M | 55 | Traditional Chinese medicine | 8 | Psoriasis |
| Biao Zhang et al | 2010 | China | M | 20 | Traditional Chinese medicine | 2 | Psoriasis |
| Biao Zhang et al | 2010 | China | M | 26 | Traditional Chinese medicine | 2 | Psoriasis |
| Yiqun Jiang et al | 2009 | China | F | 36 | Traditional Chinese medicine | UK | Psoriasis |
| Lingang Liu et al | 2009 | China | M | 19 | Traditional Chinese medicine | 2 | Psoriasis |
| Hua Du et al | 2009 | China | M | 10 | Traditional Chinese medicine | 2 | Epilepsy |
| Shaoping Yang et al | 2009 | China | M | 21 | Traditional Chinese medicine | 3 | Epilepsy |
| Tao Huang et al | 2009 | China | F | 59 | Traditional Chinese medicine | 1 | Rheumatoid arthritis |
| Rongying Sun et al | 2008 | China | F | 36 | Traditional Chinese medicine | 7 | Psoriasis |
| Yongxia Dai et al | 2008 | China | M | 48 | Traditional Chinese medicine | 5 | Psoriasis |
| Limin Li et al | 2008 | China | M | 59 | Traditional Chinese medicine | 4 | Psoriasis |
| Yali He et al | 2007 | China | M | 33 | Traditional Chinese medicine | 1 | Epilepsy |
| Yucheng Huang et al | 2007 | China | M | 55 | Traditional Chinese medicine | 1 | Psoriasis |
| N. M Hanjani et al | 2007 | USA | F | 42 | Traditional Chinese medicine | UK | Myeloid leukemia |
| Chunguang Tong et al | 2006 | China | M | 38 | Traditional Chinese medicine | 20 | Psoriasis |
| Renqi Lu et al | 2006 | China | F | 37 | Traditional Chinese medicine | 7 | Psoriasis |
| Zhenlu Li et al | 2006 | China | M | 36 | Traditional Chinese medicine | 1 | Psoriasis |
| Zhenlu Li et al | 2006 | China | M | 55 | Traditional Chinese medicine | 0.25 | Psoriasis |
| Yanhui Chen et al | 2006 | China | M | 42 | Traditional Chinese medicine | 6 | Psoriasis |
| Jiangan Zhang et al | 2006 | China | M | 54 | Traditional Chinese medicine | 10 | Epilepsy |
| Bingqing Cao et al | 2005 | China | F | 29 | Traditional Chinese medicine | 7 | Constipation |
| Xueli Fan et al | 2005 | China | M | 40 | Traditional Chinese medicine | UK | Psoriasis |
| Jingyan He et al | 2005 | China | M | 48 | Traditional Chinese medicine | 2 | Psoriasis |
| Jiang Jin et al | 2005 | China | F | 23 | Traditional Chinese medicine | 8 | Constipation |
| Jiang Jin et al | 2005 | China | M | 19 | Traditional Chinese medicine | 0.5 | Epilepsy |
| Yanning Wang et al | 2003 | China | M | 22 | Traditional Chinese medicine | 0.33 | Psoriasis |
| Weijie Cao et al | 2002 | China | F | 28 | Traditional Chinese medicine | 1 | Vitiligo |
| Shuzun Yuan et al | 2002 | China | F | 29 | Traditional Chinese medicine | 0.5 | Psoriasis |
| Wei Jiang et al | 2002 | China | F | 34 | Traditional Chinese medicine | 20 | Psoriasis |
| Wei Jiang et al | 2002 | China | F | 29 | Traditional Chinese medicine | 3 | Psoriasis |
| Wei Jiang et al | 2002 | China | M | 52 | Traditional Chinese medicine | 20 | Psoriasis |
| Guoju Tu et al | 1998 | China | M | 63 | Occupational exposure | 15 | No |
| Guizhi Yang et al | 1997 | China | F | 55 | Arsenic pesticide poisoning | Once | No |
| Guizhi Yang et al | 1997 | China | M | 58 | Arsenic pesticide poisoning | Once | No |
| Li Li et al | 1996 | China | M | 67 | Traditional Chinese medicine | 24 | Psoriasis |
| Ning Li et al | 1994 | China | M | 49 | Traditional Chinese medicine | UK | Epilepsy |

M: Male; F: Female; UK: unknown

STable 2: The clinical manifestations, treatment and outcomes of patients

| Author | Skin manifestations | Tumors | Treatment | | | | Time of follow-up | Outcomes |  |
| --- | --- | --- | --- | --- | --- | --- | --- | --- | --- |
|  |  |  | Remove arsenic treatment | Systemic treatment | Topical treatment | Physiotherapy |  |  |  |
| This paper | hyperkeratotic papules | No | No | Acitretin Capsules | No | No | 9 months | Keratinizing skin lesions improved |  |
| Manli Sun et al | ①hyperkeratotic papules  ②hyperpigmentation  ③hypopigmentation | No | Sodium dimercaptopropyl sulfonate | No | 0.1% Tretinoin Ointment | No | 2 weeks | pigmentation lesions improved |  |
| Weiyi Li et al | ①hyperkeratotic papules  ②hyperpigmentation  ③tumors | ①SCC  ②Bowen | No | No | No | No | No | Tumor subsided |  |
| Juanjuan Wang et al | ①hyperkeratotic papules  ②hyperpigmentation  ③hypopigmentation | No | Sodium dimercaptopropyl sulfonate | Acitretin Capsules | 0.1% Tretinoin Ointment | No | 2 months | Keratinizing and pigmentation lesions improved |  |
| Zhaorui Liu et al | ①hyperkeratotic papules  ②hyperpigmentation  ③tumors | ①SCC  ②Bowen | No | Acitretin Capsules | Imiquimed cream | operation | 3 months | Skin lesions improved |  |
| Xianxu Yang et al | ①hyperkeratotic papules  ②hyperpigmentation  ③hypopigmentation  ④tumors | Bowen | No | No | No | operation | No | Tumor subsided without metastasis |  |
| Cuicui Tian et al | hyperkeratotic papules | ①SCC  ②Bowen | No | Retinoid, hydroxychlor- oquine | 5-fluorouracil | No | 1 year | Skin lesions improved |  |
| Cuicui Tian et al | ①hyperkeratotic papules  ②tumors | ①SCC  ②Bowen | No | retinoid | No | PDT | 6 months | Tumors subsided |  |
| Cuicui Tian et al | ①hyperkeratotic papules  ②tumors | SCC | No | No | No | No | No | UK |  |
| Ying Li et al | ①hyperkeratotic papules  ②hyperpigmentation  ③hypopigmentation  ④tumors | SCC | sodium hyposulfite | Acitretin Capsules | No | ①PDT  ②operation | No | Keratinizing skin lesions improved |  |
| Qifeng Wang et al | ①hyperkeratotic papules  ②hyperpigmentation  ③hypopigmentation  ④tumors | BCC | No | Acitretin Capsules | Imiquimed cream | operation | 2 months | ①Keratinizing skin lesions improved  ②Tumor subsided |  |
| Zaiyong Wu et al | ①hyperkeratotic papules  ②hyperpigmentation  ③hypopigmentation  ④tumors | ①SCC  lymphatic metastasis  ②Bowen | No | No | No | operation | No | UK |  |
| Bingmei Liu et al | ①hyperkeratotic papules  ②hyperpigmentation  ③hypopigmentation | No | NK | NK | NK | NK | No | UK |  |
| Bingmei Liu et al | ①hyperkeratotic papules  ②hyperpigmentation  ③hypopigmentation | No | NK | NK | NK | NK | No | UK |  |
| Jingqiu Zhang et al | ①hyperkeratotic papules  ②hyperpigmentation  ③hypopigmentation | No | No | ①Vitamin C  ②Acitretin Capsules | Urea Ointment | cryotherapy | UK | Keratinized skin lesions improved |  |
| Yanping Huang et al | ①hyperkeratotic papules  ②hyperpigmentation  ③hypopigmentation  ④tumors | Bowen | No | Acitretin Capsules | No | operation | No | UK |  |
| Yang Li et al | ①hyperkeratotic papules  ②hyperpigmentation  ③tumors | SCC | No | Acitretin Capsules | No | ①operation  ②radiotherapy | 1.5months | Keratinized skin lesions improved |  |
| Xiaoqin Yi et al | ①hyperkeratotic papules  ②hyperpigmentation  ③hypopigmentation  ④tumors | SCC | No | Acitretin Capsules | No | operation | 2 months | ①Keratinizing and pigmentation lesions improved  ②Tumor subsided |  |
| Hongguo Li et al | ①hyperkeratotic papules  ②hyperpigmentation  ③tumors | Bowen | No | No | No | operation | No | UK |  |
| Jianfeng Yang et al | ①hyperkeratotic papules  ②hyperpigmentation  ③tumors | SCC | No | Acitretin Capsules | No | operation | 1.5months | ①Keratinizing lesions improved  ②Tumor subsided |  |
| Xiuling Liu et al | ①hyperkeratotic papules  ②hyperpigmentation  ③tumors | ①SCC  ②Bowen | No | Acitretin Capsules | No | operation | UK | Tumor subsided without metastasis |  |
| Xiaowen Chen et al | ①hyperkeratotic papules  ②hyperpigmentation  ③tumors | SCC | Sodium dimercaptopropyl sulfonate | No | No | operation | No | UK |  |
| Xiaowen Chen et al | ①hyperkeratotic papules  ②hyperpigmentation  ③hypopigmentation  ④tumors | SCC | Sodium dimercaptopropyl sulfonate | No | No | operation | No | UK |  |
| Xiaowen Chen et al | ①hyperkeratotic papules  ②hyperpigmentation  ③hypopigmentation  ④tumors | SCC | Sodium dimercaptopropyl sulfonate | No | No | operation | No | UK |  |
| Sijing Zhou et al | ①hyperkeratotic papules  ②hyperpigmentation  ③Mees' lines in nails | No | Sodium dimercaptopropyl sulfonate | No | No | No | 1month | Skin lesions improved |  |
| Sijing Zhou et al | hyperpigmentation | No | Sodium dimercaptopropyl sulfonate | No | No | No | No | UK |  |
| Yajuan Ji et al | ①hyperkeratotic papules  ②hyperpigmentation  ③hypopigmentation | No | No | No | No | No | 3 months | Pigmentation lesions improved |  |
| Rina Wu et al | ①hyperkeratotic papules  ②hyperpigmentation  ③tumors | SCC | No | No | No | No | No | UK |  |
| Xiaojuan Nie et al | ①hyperkeratotic papules  ②hyperpigmentation  ③hypopigmentation  ④tumors | Bowen | UK | No | No | operation | No | UK |  |
| Cuiping Gao et al | ①hyperkeratotic papules  ②hyperpigmentation  ③hypopigmentation | No | No | Other herbs | VE cream | No | UK | Skin lesions improved |  |
| Hua Zhang et al | ①hyperkeratotic papules  ②hyperpigmentation  ③hypopigmentation  ④tumors | SCC | No | Acitretin Capsules | No | Radiotherapy | No | UK |  |
| Jielin Zhang et al | ①hyperkeratotic papules  ②hyperpigmentation  ③hypopigmentation | No | sodium hyposulfite | Vitamin C | vaseline | No | 1month | Skin lesions improved |  |
| Wei Zhao et al | ①hyperkeratotic papules  ②hyperpigmentation | No | UK | UK | UK | UK | No | UK |  |
| Yuping Chen et al | ①hyperkeratotic papules  ②hyperpigmentation  ③ tumors | Bowen |  | Isotretinoin | 5- Fluorouracil | cryotherapy | No | UK |  |
| Ruili Zhang et al | ①hyperkeratotic papules  ②hyperpigmentation  ③hypopigmentation  ④tumors | ①SCC  ②Bowen | No | No | No | No | No | UK |  |
| Jun Wang et al | ①hyperkeratotic papules  ②hyperpigmentation  ③hypopigmentation  ④tumors | SCC | No | No | No | No | No | UK |  |
| 28Biao Zhang et al | ①hyperkeratotic papules  ②hyperpigmentation  ③hypopigmentation | No | Sodium dimercaptopropyl sulfonate | No | No | No | No | UK |  |
| Biao Zhang et al | ①hyperkeratotic papules  ②hyperpigmentation  ③hypopigmentation | No | Sodium dimercaptopropyl sulfonate | No | No | No | No | UK |  |
| Xiaoxia Liu et al | hyperkeratotic papules | SCC | No | No | No | No | No | UK |  |
| Wei Zhu et al | ①hyperkeratotic papules  ②hyperpigmentation  ③hypopigmentation | No | sodium hyposulfite | No | Tretinoin Ointment | No | 1month | Skin lesions improved |  |
| Guangcheng Gao et al | ①hyperkeratotic papules  ②hyperpigmentation  ③tumors | SCC  oral metastasis | No | No | No | Operation | NA | Death |  |
| Yuan Lu et al | ①hyperkeratotic papules  ②hyperpigmentation  ③hypopigmentation | No | No | No | No | No | No | UK |  |
| Jin Wei et al | ①hyperkeratotic papules  ②hyperpigmentation  ③hypopigmentation  ④tumors | SCC | No | No | No | operation  radiotherapy | No | UK |  |
| Biao Zhang et al | ①hyperkeratotic papules  ②hyperpigmentation  ③hypopigmentation | No | Sodium dimercaptopropyl sulfonate | Acitretin Capsules | No | No | No | UK |  |
| Biao Zhang et al | ①hyperkeratotic papules  ②hyperpigmentation  ③hypopigmentation | No | Sodium dimercaptopropyl sulfonate | No | No | No | No | UK |  |
| Yiqun Jiang et al | ①hyperkeratotic papules  ②hyperpigmentation  ③hypopigmentation  ④tumors | SCC | No | Acitretin Capsules | No | radiotherapy | 4 months | Skin lesions improved |  |
| Lingang Liu et al | ①hyperkeratotic papules  ②hyperpigmentation | No | Sodium dimercaptopropyl sulfonate | No | Tretinoin Ointment | No | UK | Skin lesions improved |  |
| Hua Du et al | ①hyperkeratotic papules  ②hyperpigmentation | No | No | Vitamin AD | 5% unguentum acidi salicylici  15% Urea Ointment | No | 6 months | Keratinizing lesions improved |  |
| Shaoping Yang et al | ①hyperkeratotic papules  ②hyperpigmentation  ③hypopigmentation | No | sodium hyposulfite | No | 0.1% Tretinoin Ointment | No |  | UK |  |
| Tao Huang et al | ①hyperkeratotic papules  ②hyperpigmentation  ③hypopigmentation  ④tumors | ①SCC  ②Bowen | No | No | Tretinoin Ointment | ①Mohs operation  ②PDT | 1 year | ①Keratinizing lesions improved  ②Tumor subsided |  |
| Rongying Sun et al | ①hyperkeratotic papules  ②hyperpigmentation | No | UK | UK | UK | UK | No | UK |  |
| Yongxia Dai et al | ①hyperkeratotic papules  ②tumors | Bowen | No | No | Imiquimed cream | No | UK | Tumor lesions improved |  |
| Limin Li et al | ①hyperkeratotic papules  ②hyperpigmentation  ③hypopigmentation  ④tumors | SCC | UK | UK | UK | UK | No | UK |  |
| Yali He et al | ①hyperkeratotic papules  ②hyperpigmentation  ③hypopigmentation | No | UK | UK | UK | UK | No | UK |  |
| Yucheng Huang et al | ①hyperkeratotic papules  ②hyperpigmentation | No | Sodium dimercaptopropyl sulfonate | No | No | No | 1 month | Skin lesions improved |  |
| N. M Hanjani et al | ①hyperkeratotic papules  ②hyperpigmentation  ③Mees' lines in nails | No | No | No | No | No | UK | UK |  |
| Chunguang Tong et al | ①hyperkeratotic papules  ②hyperpigmentation  ③hypopigmentation  ④tumors | BCC | Sodium dimercaptopropyl sulfonate | No | No | operation  cryotherapy | 1 month | Skin lesions improved |  |
| Renqi Lu et al | ①hyperkeratotic papules  ②hyperpigmentation  ③tumors | SCC | No | Acitretin Capsules | No | No | 2 months | Keratinizing and pigmentation lesions improved |  |
| Zhenlu Li et al | ①hyperkeratotic papules  ②hyperpigmentation  ③hypopigmentation | No | UK | UK | UK | UK | No | UK |  |
| Zhenlu Li et al | ①hyperkeratotic papules  ②hyperpigmentation  ③tumors | SCC | No | No | No | radiotherapy | 5 years | Tumors subsided |  |
| Yanhui Chen et al | ①hyperkeratotic papules  ②hyperpigmentation  ③hypopigmentation | No | No | Acitretin Capsules | Tretinoin Ointment | No | 1month | Skin lesions improved |  |
| Jiangan Zhang et al | ①hyperkeratotic papules  ②hyperpigmentation  ③hypopigmentation  ④tumors | Bowen | No | Acitretin Capsules | No | operation | No | UK |  |
| Bingqing Cao et al | ①hyperkeratotic papules  ②hyperpigmentation  ③hypopigmentation | No | sodium hyposulfite | No | keratolytics | No | UK | Skin lesions improved |  |
| Xueli Fan et al | ①hyperkeratotic papules  ②hyperpigmentation  ③tumors | SCC | UK | UK | UK | UK | No | UK |  |
| Jingyan He et al | ①hyperkeratotic papules  ②hyperpigmentation  ③Mees' lines in nails  ④tumors | ①SCC  ②Bowen | UK | UK | UK | UK | No | UK |  |
| Jiang Jin et al | ①hyperkeratotic papules  ②hyperpigmentation  ③hypopigmentation | No | UK | UK | UK | UK | No | UK |  |
| Jiang Jin et al | ①hyperkeratotic papules  ②hyperpigmentation  ③hypopigmentation | No | sodium hyposulfite | No | No | No | 2 weeks | Skin lesions improved |  |
| Yanning Wang et al | ①hyperkeratotic papules  ②hyperpigmentation | No | sodium hyposulfite | Other herbs | Tretinoin Ointment | No | No | UK |  |
| Weijie Cao et al | ①hyperkeratotic papules  ②hyperpigmentation | No | UK | No | No | No | UK | Skin lesions improved |  |
| Shuzun Yuan et al | ①hyperkeratotic papules  ②hyperpigmentation  ③hypopigmentation | No | sodium hyposulfite | Vitamin C | No | No | 2 weeks | Keratinizing and pigmentation lesions improved |  |
| Wei Jiang et al | ①hyperkeratotic papules  ②hyperpigmentation  ③hypopigmentation | No | UK | UK | UK | UK | No | UK |  |
| Wei Jiang et al | ①hyperkeratotic papules  ②hyperpigmentation  ③hypopigmentation | No | UK | UK | UK | UK | No | UK |  |
| Wei Jiang et al | ①hyperkeratotic papules  ②hyperpigmentation  ③hypopigmentation | No | UK | UK | UK | UK | No | UK |  |
| Guoju Tu et al | ①hyperpigmentation  ②tumors | SCC | No | No | No | radiotherapy | 2 months | ①Tumors subsided  ②Improvement of skin lesions was not obvious |  |
| Guizhi Yang et al | ①hyperkeratotic papules | Bowen | No | No | 5- Fluorouracil | cryotherapy | 3months | Tumors subsided |  |
| Guizhi Yang et al | ①hyperkeratotic papules | No | No | No | 5- Fluorouracil | No | No | UK |  |
| Li Li et al | ①hyperkeratotic papules  ②tumors | SCC  bone metastases | UK | UK | UK | UK | No | UK |  |
| Ning Li et al | ①hyperkeratotic papules  ②Mees' lines in nails  ③tumors | ①SCC  ②Bowen | UK | UK | UK | UK | No | UK |  |

UK: Unknown
